# Methyl 2-(benzene­sulfonamido)acetate

**DOI:** 10.1107/S160053680901616X

**Published:** 2009-05-07

**Authors:** Muhammad Nadeem Arshad, Islam Ullah Khan, Muhammad Zia-ur-Rehman, Muhammad Shafiq

**Affiliations:** a Department of Chemistry, Government College University, Lahore 54000, Pakistan; b Applied Chemistry Research Centre, PCSIR Laboratories Complex, Lahore 54600, Pakistan

## Abstract

The title compound, C_9_H_11_NO_4_S, is of inter­est as a precursor to biologically active benzothia­zines. The crystal structure is stabilized by inter­molecular N—H⋯O and C—H⋯O inter­actions.

## Related literature

For the synthesis and biological evaluation of sulfur-containing heterocyclic compounds, see: Zia-ur-Rehman *et al.* (2005 ▶, 2006 ▶, 2009 ▶); Xiao & Timberlake (2000 ▶); Martinez *et al.* (2000 ▶); Berredjem *et al.* (2000 ▶); Lee & Lee (2002 ▶). For related literature on sulfonamides, see: Esteve & Bidal (2002 ▶); Soledade *et al.* (2006 ▶). For related structures, see: Gowda *et al.* (2007*a* ▶,*b* ▶,*c* ▶).
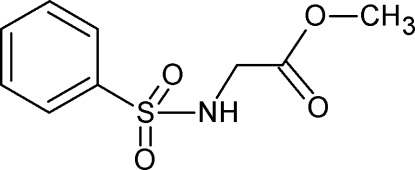


## Experimental

### 

#### Crystal data

C_9_H_11_NO_4_S
*M* *_r_* = 229.26
Monoclinic, 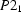

*a* = 9.7268 (8) Å
*b* = 5.0781 (4) Å
*c* = 10.9286 (9) Å
β = 100.087 (3)°
*V* = 531.46 (7) Å^3^
*Z* = 2
Mo *K*α radiation
μ = 0.30 mm^−1^
*T* = 296 K
0.23 × 0.11 × 0.08 mm

#### Data collection

Bruker APEXII CCD area-detector diffractometer
Absorption correction: multi-scan (*SADABS*; Bruker, 2007 ▶) *T*_min_ = 0.935, *T*_max_ = 0.977
6159 measured reflections
2216 independent reflections
1944 reflections with *I* > 2σ(*I*)
*R*_int_ = 0.028

#### Refinement

*R*[*F* ^2^ > 2σ(*F* ^2^)] = 0.036
*wR*(*F* ^2^) = 0.092
*S* = 1.07
2216 reflections
137 parameters
1 restraint
H-atom parameters constrained
Δρ_max_ = 0.25 e Å^−3^
Δρ_min_ = −0.26 e Å^−3^
Absolute structure: Flack (1983 ▶), 394 Friedel pairs
Flack parameter: 0.089 (8)

### 

Data collection: *APEX2* (Bruker, 2007 ▶); cell refinement: *SAINT* (Bruker, 2007 ▶); data reduction: *SAINT*; program(s) used to solve structure: *SHELXS97* (Sheldrick, 2008 ▶); program(s) used to refine structure: *SHELXL97* (Sheldrick, 2008 ▶); molecular graphics: *PLATON* (Spek, 2009 ▶) and *Mercury* (Macrae *et al.*, 2006 ▶); software used to prepare material for publication: *SHELXTL* (Sheldrick, 2008 ▶) and local programs.

## Supplementary Material

Crystal structure: contains datablocks I, global. DOI: 10.1107/S160053680901616X/bt2940sup1.cif

Structure factors: contains datablocks I. DOI: 10.1107/S160053680901616X/bt2940Isup2.hkl

Additional supplementary materials:  crystallographic information; 3D view; checkCIF report

## Figures and Tables

**Table 1:** Hydrogen-bond geometry (Å, °)

*D*—H⋯*A*	*D*—H	H⋯*A*	*D*⋯*A*	*D*—H⋯*A*
N1—H1⋯O3^i^	0.86	2.28	2.998 (2)	141
C7—H7*B*⋯O3^ii^	0.97	2.55	3.503 (4)	168

Symmetry codes: (i) 
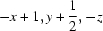
; (ii) 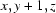.

**Fig. 1.**
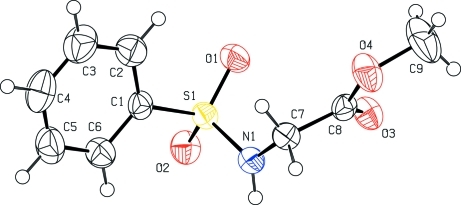
The molecular structure of the title compound showing the atom labelling scheme. Displacement ellipsoids are drawn at the 50% probability level.

**Fig. 2.**
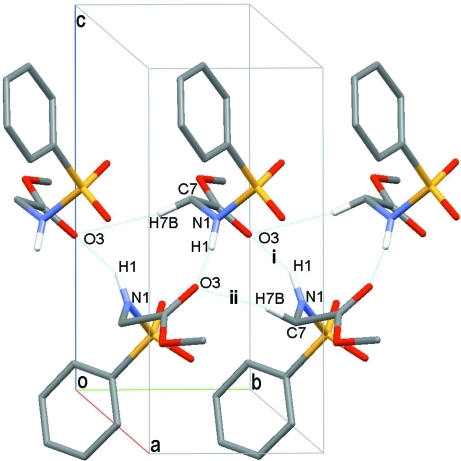
Part of the crystal structure, showing hydrogen bond interactions (dashed lines). along the [0 0 1] direction. H atoms not involved in hydrogen bonding have been omitted for clarity.

## supplementary crystallographic information

### Comment

Sulfonamide is an important functionality found in many naturally occurring as well as synthetic compounds which possess numerous types of biological activities (Soledade *et al.*, 2006; Esteve & Bidal, 2002; Xiao & Timberlake, 2000; Martinez *et al.*, 2000; Berredjem *et al.*, 2000; Lee & Lee, 2002). In the present paper, the structure of the title compound has been determined as a part of our ongoing research on the synthesis and biological evaluation of sulfur containing heterocyclic compounds (Zia-ur-Rehman *et al.*, 2005, 2006, 2009). In the molecule of (I) (Fig. 1), bond lengths and bond angles are almost similar to those in related sulfonamide molecules (Gowda *et al.*, 2007*a*, 2007*b*, 2007*c*) and the bond lengths are within normal ranges. In the crystal structure, each molecule is linked to an adjacent one through C7—H7B···O3 contacts giving rise to chains along *b*-axis. Each molecule of the chain is further linked to the one of its neighbouring chain along *a* through intermolecular N—H···O interactions.

### Experimental

A mixture of benzene sulfonic acid (4.14 g, 23.44 mmoles), glycine methyl ester hydrochloride (2.94 g, 23.44 mmol.) and distilled water (50.0 ml) was stirred for  half an hour. pH of the reaction mixture was adjusted to 8.0 with an aqueous sodium carbonate solution. After completion of the reaction, a white solid product was isolated, washed, dried and recrystallized in methanol to get the crystals suitable for for X-ray studies; m.p. 332 K.

### Refinement

H atoms  were placed in geometric positions (C—H   = 0.93-0.97 Å; N—H   = 0.86 Å) using a riding model with *U*_iso_(H) = 1.2 *U*_eq_(C,N) or *U*_iso_(H) = 1.5 *U*_eq_(C_methyl_).

### Crystal data

**Table:**

C_9_H_11_NO_4_S	*F*(000) = 240
*M**_r_* = 229.26	*D*_x_ = 1.433 Mg m^−^^3^
Monoclinic, *P*2_1_	Melting point: 332 K
Hall symbol: P 2yb	Mo *K*α radiation, λ = 0.71073 Å
*a* = 9.7268 (8) Å	Cell parameters from 2710 reflections
*b* = 5.0781 (4) Å	θ = 2.6–26.6°
*c* = 10.9286 (9) Å	µ = 0.30 mm^−^^1^
β = 100.087 (3)°	*T* = 296 K
*V* = 531.46 (7) Å^3^	Plates, colourless
*Z* = 2	0.23 × 0.11 × 0.08 mm

### Data collection

**Table:**

Bruker APEXII CCD area-detector diffractometer	2216 independent reflections
Radiation source: fine-focus sealed tube	1944 reflections with *I* > 2σ(*I*)
graphite	*R*_int_ = 0.028
φ and ω scans	θ_max_ = 29.3°, θ_min_ = 2.6°
Absorption correction: multi-scan (*SADABS*; Bruker, 2007)	*h* = −12→13
*T*_min_ = 0.935, *T*_max_ = 0.977	*k* = −4→6
6159 measured reflections	*l* = −11→15

### Refinement

**Table:**

Refinement on *F*^2^	Secondary atom site location: difference Fourier map
Least-squares matrix: full	Hydrogen site location: inferred from neighbouring sites
*R*[*F*^2^ > 2σ(*F*^2^)] = 0.036	H-atom parameters constrained
*wR*(*F*^2^) = 0.092	*w* = 1/[σ^2^(*F*_o_^2^) + (0.0526*P*)^2^ + 0.0105*P*] where *P* = (*F*_o_^2^ + 2*F*_c_^2^)/3
*S* = 1.07	(Δ/σ)_max_ < 0.001
2216 reflections	Δρ_max_ = 0.25 e Å^−^^3^
137 parameters	Δρ_min_ = −0.26 e Å^−^^3^
1 restraint	Absolute structure: Flack (1983), 394 Friedel pairs
Primary atom site location: structure-invariant direct methods	Flack parameter: 0.089 (8)

### Special details

**Table:**

Geometry. All e.s.d.'s (except the e.s.d. in the dihedral angle between two l.s. planes) are estimated using the full covariance matrix. The cell e.s.d.'s are taken into account individually in the estimation of e.s.d.'s in distances, angles and torsion angles; correlations between e.s.d.'s in cell parameters are only used when they are defined by crystal symmetry. An approximate (isotropic) treatment of cell e.s.d.'s is used for estimating e.s.d.'s involving l.s. planes.
Refinement. Refinement of *F*^2^ against ALL reflections. The weighted *R*-factor *wR* and goodness of fit *S* are based on *F*^2^, conventional *R*-factors *R* are based on *F*, with *F* set to zero for negative *F*^2^. The threshold expression of *F*^2^ > σ(*F*^2^) is used only for calculating *R*-factors(gt) *etc*. and is not relevant to the choice of reflections for refinement. *R*-factors based on *F*^2^ are statistically about twice as large as those based on *F*, and *R*– factors based on ALL data will be even larger.

### Fractional atomic coordinates and isotropic or equivalent isotropic displacement parameters (Å^2^)

**Table:**

	*x*	*y*	*z*	*U*_iso_*/*U*_eq_	
S1	0.61607 (5)	0.09862 (10)	0.21011 (5)	0.03580 (15)	
O1	0.52527 (16)	−0.0799 (4)	0.25831 (16)	0.0482 (4)	
O2	0.72360 (15)	0.0024 (4)	0.14799 (15)	0.0468 (4)	
O3	0.27134 (16)	0.0012 (4)	0.03143 (16)	0.0469 (4)	
O4	0.16275 (17)	0.2366 (4)	0.15812 (18)	0.0589 (5)	
N1	0.51744 (17)	0.2853 (4)	0.11147 (16)	0.0389 (5)	
H1	0.5423	0.3294	0.0426	0.047*	
C1	0.6962 (2)	0.2993 (5)	0.3344 (2)	0.0366 (5)	
C2	0.6546 (3)	0.2854 (7)	0.4486 (2)	0.0537 (7)	
H2	0.5847	0.1687	0.4614	0.064*	
C3	0.7181 (3)	0.4473 (8)	0.5441 (2)	0.0669 (9)	
H3	0.6913	0.4382	0.6216	0.080*	
C4	0.8199 (3)	0.6206 (8)	0.5249 (3)	0.0646 (8)	
H4	0.8612	0.7303	0.5891	0.078*	
C5	0.8614 (3)	0.6333 (7)	0.4113 (3)	0.0602 (7)	
H5	0.9317	0.7494	0.3992	0.072*	
C6	0.7990 (2)	0.4739 (5)	0.3148 (2)	0.0481 (6)	
H6	0.8259	0.4843	0.2374	0.058*	
C7	0.3858 (2)	0.3768 (5)	0.1419 (2)	0.0365 (5)	
H7B	0.3610	0.5437	0.1008	0.044*	
H7A	0.3966	0.4050	0.2309	0.044*	
C8	0.2709 (2)	0.1813 (5)	0.10223 (19)	0.0346 (5)	
C9	0.0435 (3)	0.0566 (10)	0.1303 (3)	0.0886 (12)	
H9A	0.0132	0.0469	0.0419	0.133*	
H9B	−0.0315	0.1204	0.1687	0.133*	
H9C	0.0709	−0.1155	0.1619	0.133*	

### Atomic displacement parameters (Å^2^)

**Table:**

	*U*^11^	*U*^22^	*U*^33^	*U*^12^	*U*^13^	*U*^23^
S1	0.0316 (2)	0.0341 (3)	0.0417 (3)	−0.0008 (3)	0.00653 (19)	0.0014 (3)
O1	0.0446 (9)	0.0426 (10)	0.0565 (10)	−0.0084 (8)	0.0069 (8)	0.0094 (9)
O2	0.0390 (8)	0.0496 (11)	0.0525 (10)	0.0075 (8)	0.0099 (7)	−0.0064 (8)
O3	0.0415 (9)	0.0443 (10)	0.0559 (10)	−0.0052 (8)	0.0114 (7)	−0.0095 (9)
O4	0.0329 (8)	0.0774 (14)	0.0701 (11)	−0.0025 (9)	0.0194 (8)	−0.0181 (11)
N1	0.0333 (9)	0.0445 (12)	0.0393 (10)	−0.0014 (9)	0.0074 (8)	0.0040 (9)
C1	0.0292 (10)	0.0377 (13)	0.0412 (11)	0.0037 (9)	0.0014 (8)	0.0020 (10)
C2	0.0461 (14)	0.0662 (19)	0.0502 (14)	−0.0020 (14)	0.0123 (11)	−0.0009 (14)
C3	0.0632 (17)	0.091 (3)	0.0467 (15)	0.0050 (18)	0.0092 (13)	−0.0126 (16)
C4	0.0643 (16)	0.064 (2)	0.0587 (15)	0.0052 (18)	−0.0098 (12)	−0.0190 (18)
C5	0.0537 (14)	0.051 (2)	0.0701 (16)	−0.0110 (15)	−0.0044 (12)	−0.0030 (16)
C6	0.0469 (13)	0.0451 (16)	0.0518 (14)	−0.0052 (12)	0.0071 (11)	0.0017 (12)
C7	0.0352 (11)	0.0327 (13)	0.0423 (11)	0.0031 (10)	0.0082 (9)	0.0019 (10)
C8	0.0295 (10)	0.0394 (14)	0.0342 (10)	0.0059 (9)	0.0038 (8)	0.0031 (10)
C9	0.0397 (14)	0.116 (3)	0.114 (3)	−0.0180 (19)	0.0257 (16)	−0.015 (3)

### Geometric parameters (Å, °)

**Table:**

S1—O1	1.4290 (16)	C3—C4	1.369 (4)
S1—O2	1.4291 (15)	C3—H3	0.9300
S1—N1	1.618 (2)	C4—C5	1.372 (4)
S1—C1	1.767 (2)	C4—H4	0.9300
O3—C8	1.198 (3)	C5—C6	1.383 (4)
O4—C8	1.336 (2)	C5—H5	0.9300
O4—C9	1.466 (4)	C6—H6	0.9300
N1—C7	1.454 (3)	C7—C8	1.501 (3)
N1—H1	0.8600	C7—H7B	0.9700
C1—C2	1.380 (3)	C7—H7A	0.9700
C1—C6	1.381 (3)	C9—H9A	0.9600
C2—C3	1.386 (4)	C9—H9B	0.9600
C2—H2	0.9300	C9—H9C	0.9600
			
O1—S1—O2	120.61 (11)	C4—C5—C6	120.2 (3)
O1—S1—N1	106.53 (9)	C4—C5—H5	119.9
O2—S1—N1	106.36 (9)	C6—C5—H5	119.9
O1—S1—C1	107.48 (10)	C1—C6—C5	119.4 (2)
O2—S1—C1	107.55 (10)	C1—C6—H6	120.3
N1—S1—C1	107.73 (10)	C5—C6—H6	120.3
C8—O4—C9	115.6 (2)	N1—C7—C8	111.31 (18)
C7—N1—S1	118.54 (14)	N1—C7—H7B	109.4
C7—N1—H1	120.7	C8—C7—H7B	109.4
S1—N1—H1	120.7	N1—C7—H7A	109.4
C2—C1—C6	120.6 (2)	C8—C7—H7A	109.4
C2—C1—S1	120.4 (2)	H7B—C7—H7A	108.0
C6—C1—S1	119.01 (17)	O3—C8—O4	123.2 (2)
C1—C2—C3	119.2 (3)	O3—C8—C7	127.20 (19)
C1—C2—H2	120.4	O4—C8—C7	109.60 (19)
C3—C2—H2	120.4	O4—C9—H9A	109.5
C4—C3—C2	120.3 (3)	O4—C9—H9B	109.5
C4—C3—H3	119.8	H9A—C9—H9B	109.5
C2—C3—H3	119.8	O4—C9—H9C	109.5
C3—C4—C5	120.3 (3)	H9A—C9—H9C	109.5
C3—C4—H4	119.9	H9B—C9—H9C	109.5
C5—C4—H4	119.9		
			
O1—S1—N1—C7	40.2 (2)	C1—C2—C3—C4	0.6 (5)
O2—S1—N1—C7	170.05 (17)	C2—C3—C4—C5	−0.8 (5)
C1—S1—N1—C7	−74.87 (19)	C3—C4—C5—C6	1.0 (5)
O1—S1—C1—C2	−7.1 (2)	C2—C1—C6—C5	0.8 (4)
O2—S1—C1—C2	−138.4 (2)	S1—C1—C6—C5	179.5 (2)
N1—S1—C1—C2	107.3 (2)	C4—C5—C6—C1	−1.0 (4)
O1—S1—C1—C6	174.20 (18)	S1—N1—C7—C8	−86.9 (2)
O2—S1—C1—C6	42.9 (2)	C9—O4—C8—O3	1.7 (3)
N1—S1—C1—C6	−71.3 (2)	C9—O4—C8—C7	−178.5 (2)
C6—C1—C2—C3	−0.6 (4)	N1—C7—C8—O3	−15.8 (3)
S1—C1—C2—C3	−179.2 (2)	N1—C7—C8—O4	164.42 (18)

### Hydrogen-bond geometry (Å, °)

**Table:**

*D*—H···*A*	*D*—H	H···*A*	*D*···*A*	*D*—H···*A*
N1—H1···O3^i^	0.86	2.28	2.998 (2)	141
C7—H7B···O3^ii^	0.97	2.55	3.503 (4)	168

Symmetry codes: (i) −*x*+1, *y*+1/2, −*z*; (ii) *x*, *y*+1, *z*.
